# Recruitment and training of alveolar macrophages after pneumococcal pneumonia

**DOI:** 10.1172/jci.insight.150239

**Published:** 2022-03-08

**Authors:** Emad I. Arafa, Anukul T. Shenoy, Kimberly A. Barker, Neelou S. Etesami, Ian M.C. Martin, Carolina Lyon De Ana, Elim Na, Christine V. Odom, Wesley N. Goltry, Filiz T. Korkmaz, Alicia K. Wooten, Anna C. Belkina, Antoine Guillon, E. Camilla Forsberg, Matthew R. Jones, Lee J. Quinton, Joseph P. Mizgerd

**Affiliations:** 1 Pulmonary Center,; 2 Department of Medicine, and; 3 Department of Microbiology, Boston University School of Medicine, Boston, Massachusetts, USA.; 4 Department of Medicine, University of Massachusetts Medical School, Worcester, Massachusetts, USA.; 5 Department of Pathology and Laboratory Medicine, and; 6 Flow Cytometry Core Facility, Boston University School of Medicine, Boston, Massachusetts, USA.; 7 CHRU of Tours, Intensive Medicine, Resuscitation Service, INSERM, Center for the Study of Respiratory Pathologies (CEPR), University of Tours, Tours, France.; 8 Institute for the Biology of Stem Cells, University of California Santa Cruz, Santa Cruz, California, USA.; 9 Department of Biochemistry, Boston University School of Medicine, Boston, Massachusetts, USA.

**Keywords:** Immunology, Pulmonology, Bacterial infections, Macrophages

## Abstract

Recovery from pneumococcal pneumonia remodels the pool of alveolar macrophages so that they exhibit new surface marker profiles, transcriptomes, metabolomes, and responses to infection. Mechanisms mediating alveolar macrophage phenotypes after pneumococcal pneumonia have not been delineated. IFN-γ and its receptor on alveolar macrophages were essential for certain, but not all, aspects of the remodeled alveolar macrophage phenotype. IFN-γ was produced by CD4^+^ T cells plus other cells, and CD4^+^ cell depletion did not prevent alveolar macrophage remodeling. In mice infected or recovering from pneumococcus, monocytes were recruited to the lungs, and the monocyte-derived macrophages developed characteristics of alveolar macrophages. CCR2 mediated the early monocyte recruitment but was not essential to the development of the remodeled alveolar macrophage phenotype. Lineage tracing demonstrated that recovery from pneumococcal pneumonias converted the pool of alveolar macrophages from being primarily of embryonic origin to being primarily of adult hematopoietic stem cell origin. Alveolar macrophages of either origin demonstrated similar remodeled phenotypes, suggesting that ontogeny did not dictate phenotype. Our data reveal that the remodeled alveolar macrophage phenotype in lungs recovered from pneumococcal pneumonia results from a combination of new recruitment plus training of both the original cells and the new recruits.

## Introduction

Lung infections are a leading cause of morbidity and mortality (1–5). Microbes that enter the air spaces of the lung encounter alveolar macrophages (AM), which function as sentinels and immune modulators. These cells protect the lung by clearing microbial and nonmicrobial agents, initiating pulmonary inflammation, and contributing to resolution and repair (6, 7).

AM can arise from either of 2 disparate origins. Initially, they derive from embryonic sources, including yolk sac precursors and fetal liver hematopoiesis (8–11). Postnatally, AM appear that derive from bone marrow hematopoiesis (12–14), with this fraction continuously expanding and encompassing ~40% of the AM pool at 1 year of age in C57BL/6 mice in specific pathogen-free environments (11). Factors that kill AM can more rapidly convert the AM pool to becoming dominated by monocyte-derived cells from adult hematopoiesis (12–14). Several pneumonia-causing microbes target macrophages for programmed cell death (15–17) and accelerate turnover of the AM pool (12–14). The AM of fetal origin and of adult hematopoietic origin are largely similar but not identical to each other when compared across multiple characteristics including surface markers, gene expression, and functional activities (18–20).

After recovery from respiratory infections, the AM pool can exhibit remodeled phenotypes (21). Recovery from adenoviral pneumonia results in AM with increased surface MHC-II and improved responses to subsequent bacterial infections including elevated induction of neutrophil chemokines (22); this remodeling occurs entirely within the initial AM pool of embryonic origin, with no replacement by monocyte-derived AM (22). The adenovirus-induced remodeling is dependent on IFN-γ derived from CD8^+^ T cells recruited to the lung during the initial adenovirus infection (22). Recovery from influenza pneumonia can result in AM with increased surface MHC-II, and improved responses to subsequent bacterial infections including elevated induction of IL-6, which requires CCR2-mediated recruitment of monocytes that differentiate and replace the AM of embryonic origin (14). These studies depict 2 contrasting paradigms for how AM remodel following pneumonia recovery, new phenotypes developing in the initial cells *vs*. replacement of initial cells by new AM bearing different phenotypes (14, 22).

Recovery from pneumococcal pneumonia results in a profoundly improved protection against heterotypic infection (i.e., by a different serotype), due to a combination of resident memory lymphocytes as well as the remodeling of AM (21, 23–26). The remodeled AM in this setting have increased surface MHC-II, decreased surface Siglec F, enhanced creatine metabolism, and profoundly altered transcriptomes both at rest and in response to infection, better matching the immune responses of human AM (21). Mechanisms by which AM remodel in pneumococcus-recovered lungs are unclear. Pneumococci can kill AM (15) and the transcriptomes of remodeled AM in pneumococcus-recovered lungs include transcript changes consistent with AM derived from adult monocytes (21), which suggests remodeling by replacement as observed after influenza pneumonia (14). Pneumococcal pneumonia elicits IFN-γ from neutrophils (27, 28) and results in IFN-γ–producing CD4^+^ T cells in the lung (23), which may contribute to AM remodeling analogously to the IFN-γ from CD8^+^ T cells during adenoviral pneumonia (22). The phenotypes of AM recovered from pneumococcal pneumonia (21) differ from those observed following adenoviral (22) or influenza (14) pneumonia, suggesting the possibility that neither paradigm may apply to the context of bacterial lung infections.

In this report, we use lineage tracing and transgenic mice to investigate the mechanisms driving AM remodeling following the recovery from pneumococcal pneumonia.

## Results

### Numbers and surface marker phenotypes of alveolar macrophages fluctuate after infection.

**Figure 1:**
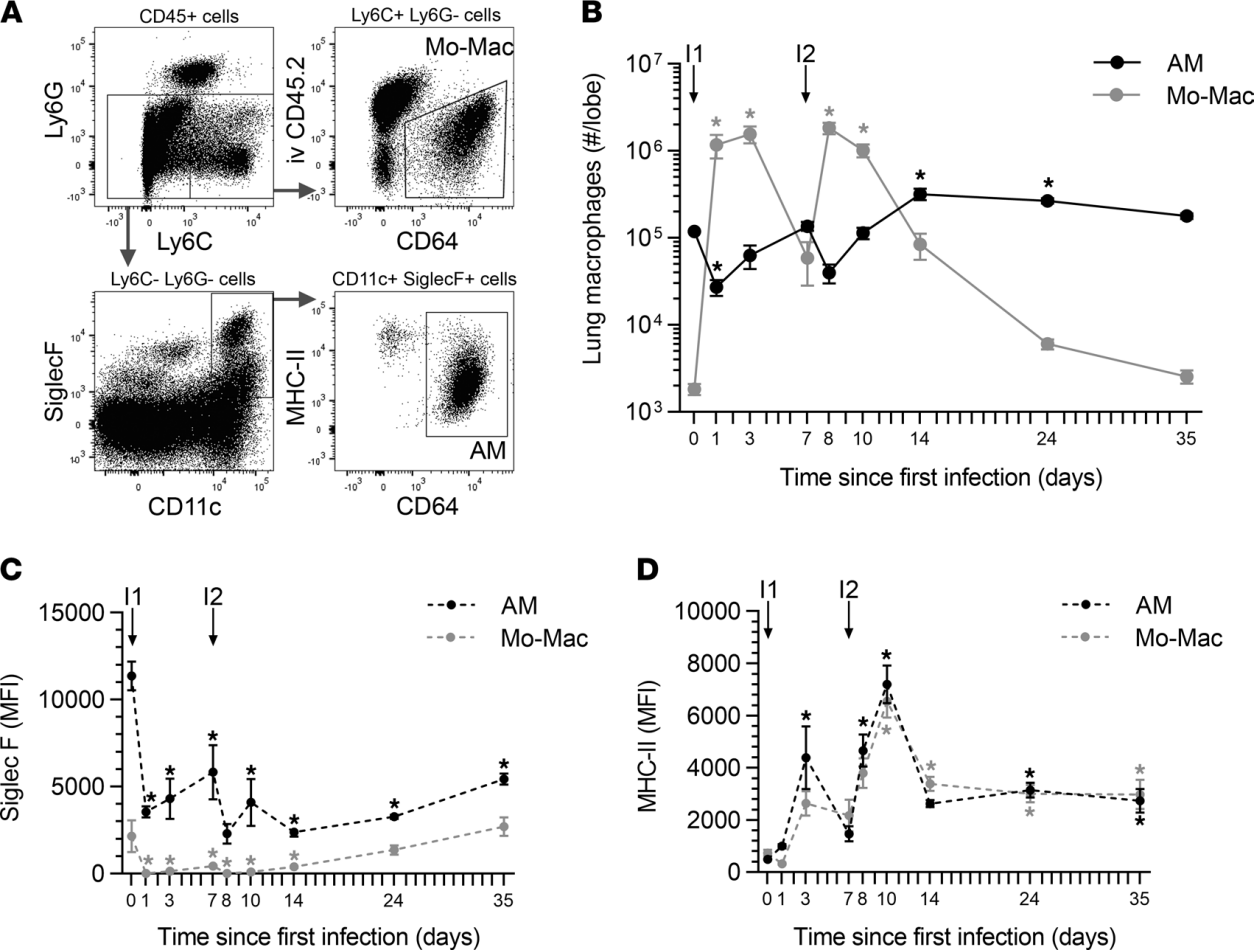
Macrophage dynamics after pneumococcal infections. Single-cell suspensions were generated from left lung lobes and processed using flow cytometry at select time points during generation of pneumococcal experience. (**A**) AM and inflammatory monocyte-macrophages (Mo-Macs) were identified as CD45^+^Ly6g^–^CD64^+^SiglecF^+^CD11c^+^ cells and CD45^+^Ly6g^–^CD64^+^SiglecF^+^Ly6c^+^ cells, respectively. Panels represent a mouse with Sp19F pneumonia 7 days after infection. (**B**) Numbers of AM and Mo-Macs were calculated from the frequency of total measured by FlowJo software. (**C** and **D**) Median fluorescent intensity (MFI) of Siglec F and MHC-II on AM and Mo-Macs at select time points after a first infection (I1) or second infection (I2) with Sp19F. Values are expressed as mean ± SEM. One-way ANOVA with Dunnett’s multiple-comparison test was used to examine significance. * *P* < 0.05 compared with day 0.

Naturally acquired immune defense against pneumococcal pneumonia can be achieved in mice by administering 2 self-limiting respiratory infections with *Streptococcus pneumoniae* serotype 19F (Sp19F) spaced 1 week apart followed by a month of recovery, after which the mice demonstrate a long-lasting heterotypic (serotype-mismatched) protection that involves remodeled AM of unknown sources (21, 23–26). To determine whether and how AM numbers change during the infection history that establishes heterotypic protection against pneumococcal pneumonia (21, 23–26), naive mice were ushered through serial infections, and at designated time points, lungs were collected and digested to single-cell suspensions (SCS) for flow cytometry. After gating on singlet cells, live cells, and CD45^+^ cells, AM were identified as Ly6G^–^Ly6C^–^CD11c^+^SiglecF^+^CD64^+^ cells (Figure 1A). AM numbers were significantly reduced by two-thirds 1 day after Sp19F challenge, but they returned to baseline between days 3 and 7 (Figure 1B). AM numbers followed a similar trend after the second pneumococcal challenge, dropping by two-thirds at day 1 but becoming restored soon after (Figure 1B). AM numbers continued to increase and were elevated at days 14 and 24 before returning toward the original baseline by day 35 (Figure 1B). These results are consistent with rapid AM losses due to pneumococcus-induced programmed cell death (15), followed by restoration resulting from recruitment and/or proliferation.

Because fully differentiated AM can be replenished from recruited monocytes (12–14), we quantified extravascular inflammatory monocyte-macrophages (Mo-Macs) throughout the time course. Mo-Macs were identified as CD45^+^Ly6G^–^Ly6C^+^CD64^+^ cells and were readily distinguished from the intravascular (stained by an anti-CD45 antibody delivered i.v.) and extravascular Ly6C^+^ cells that were CD64^–^ (Figure 1A). At baseline, there were many more AM than Mo-Macs in the lung (Figure 1B). Within 1 day after each infection, a substantial increase in Mo-Macs led to them becoming more abundant than AM in the lungs (Figure 1B). By 1 week after infection, Mo-Macs were declining in numbers in the lungs (Figure 1B). By day 35, Mo-Mac numbers returned to their initial low baseline values (Figure 1B). Thus, AM and Mo-Macs were both dynamic, with complementary kinetics. Mo-Macs increased whenever AM declined and declined when AM increased, until both returned to near baseline values by day 35.

Cardinal features of the remodeled AM in experienced lungs (recovered from prior respiratory infection) are surface marker changes, particularly decreased Siglec F and increased MHC-II (14, 18–22). We examined these surface markers on AM and Mo-Macs over the course of infections and recovery. Siglec F diminished in intensity on AM within 1 day of the first infection and never fully recovered, hovering around half the baseline levels throughout the time course and at the experienced lung time point of 35 days (Figure 1C). MHC-II was slower to change and then far more dynamic. MHC-II first increased on AM at day 3, reached peak intensity at day 10, and then established a new stable elevated set point by day 14, which was maintained through the experienced lung time point of 35 days (Figure 1D). The lung Mo-Macs demonstrated similar changes. Siglec F was low on Mo-Macs compared with AM, but this dim signal diminished due to infection (Figure 1D). MHC-II was present at similar intensities on Mo-Macs and AM and showed similar changes throughout the time course (Figure 1D). Thus, Siglec F and MHC-II display different kinetics, with the former accomplishing most of its change within a day (suggesting innate immune signaling) while the latter developed over a longer time frame, peaking at day 10 before dropping to a new baseline (more consistent with adaptive immunity signaling). Both AM and Mo-Mac responded analogously during the infection and recovery periods.

### The remodeled alveolar macrophage phenotype does not require CD4^+^ cells.

**Figure 2:**
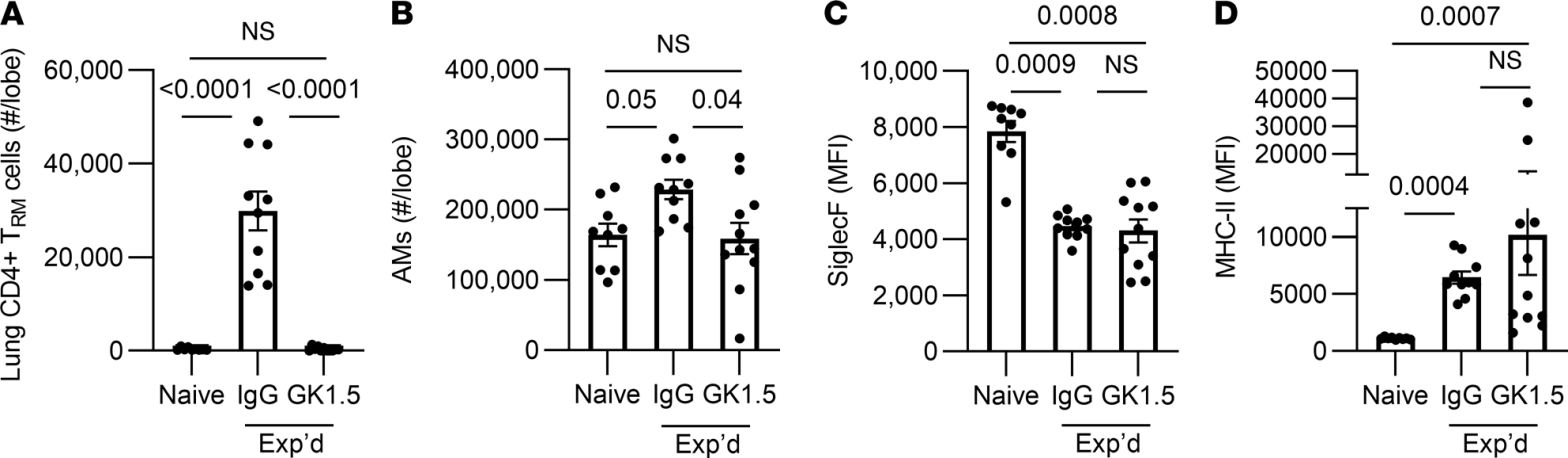
CD4^+^ cells and the experienced AM phenotype. C57BL/6 mice received i.p. and i.n. GK1.5 to deplete CD4^+^ T cells, or IgG as control, during each of the Sp19F infections — the last of which was 4 weeks prior to studies of T cells and AM in the lungs. (**A**) Enumeration of extravascular CD4^+^CD69^+^CD11a^+^CD44^+^CD62L^–^ cells per lung lobe reveals a loss of CD4^+^ resident memory T (T_RM_) cells in the GK1.5-treated group. (**B**) Enumeration of alveolar macrophages (defined as CD11c^+^SiglecF^+^CD64^+^). (**C** and **D**) Median fluorescent intensity (MFI) of Siglec F and MHC-II on AM. Values are expressed as mean ± SEM. *n* = 8–12 mice/group. Kruskal-Wallis with Dunn’s multiple-comparison tests were used to examine significance.

The AM remodeling induced by adenoviral infections, including increased MHC-II, depends on T cell–derived IFN-γ (22). Pneumococcal experienced lungs exhibit a transient recruitment of effector CD4^+^ T cells that become a population of CD4^+^ resident memory T (T_RM_) cells, and these CD4^+^ cells express IFN-γ, IL-17, and other cytokines (23–26). To test whether CD4^+^ T cells are essential for the remodeled phenotype of AM in pneumococcus-experienced lungs, mice received doses of GK1.5 antibodies to deplete CD4^+^ cells (or isotype-matched nonspecific IgG as control) throughout the initial infections, and then lungs were characterized at day 35. This GK1.5 regimen completely eliminated CD4^+^ T_RM_ cell accumulation (Figure 2A), demonstrating that this hallmark of experienced lungs requires CD4^+^ T cells during the initial infections. We examined whether CD4^+^ cell depletion had an impact on AM numbers and phenotype in experienced lungs (Figure 2, B–D). Experienced mice that had CD4^+^ cell depletion during initial rounds of infection exhibited significantly less AM numbers compared with control experienced mice (Figure 2B). Siglec F intensity on AM was significantly decreased on experienced mice, regardless of whether the mice had received the GK1.5 depletion regimen or nonspecific IgG, suggesting that the diminished Siglec F intensity on AM associated with pneumococcal experience was not dependent on adaptive immune signaling (Figure 2C). Analogously, MHC-II intensity was increased in both experienced mice that received GK1.5 depletion antibody — as well as in the experienced mice that received nonspecific IgG — in comparison with naive mice; there was comparable MHC-II intensity between the 2 groups of experienced mice (Figure 2D). Thus, CD4^+^ T cells during initial infection and CD4^+^ T_RM_ cell formation are not essential for the remodeled AM phenotype in pneumococcus-experienced lungs.

### IFN-γ is derived from diverse sources in the infected lung.

**Figure 3:**
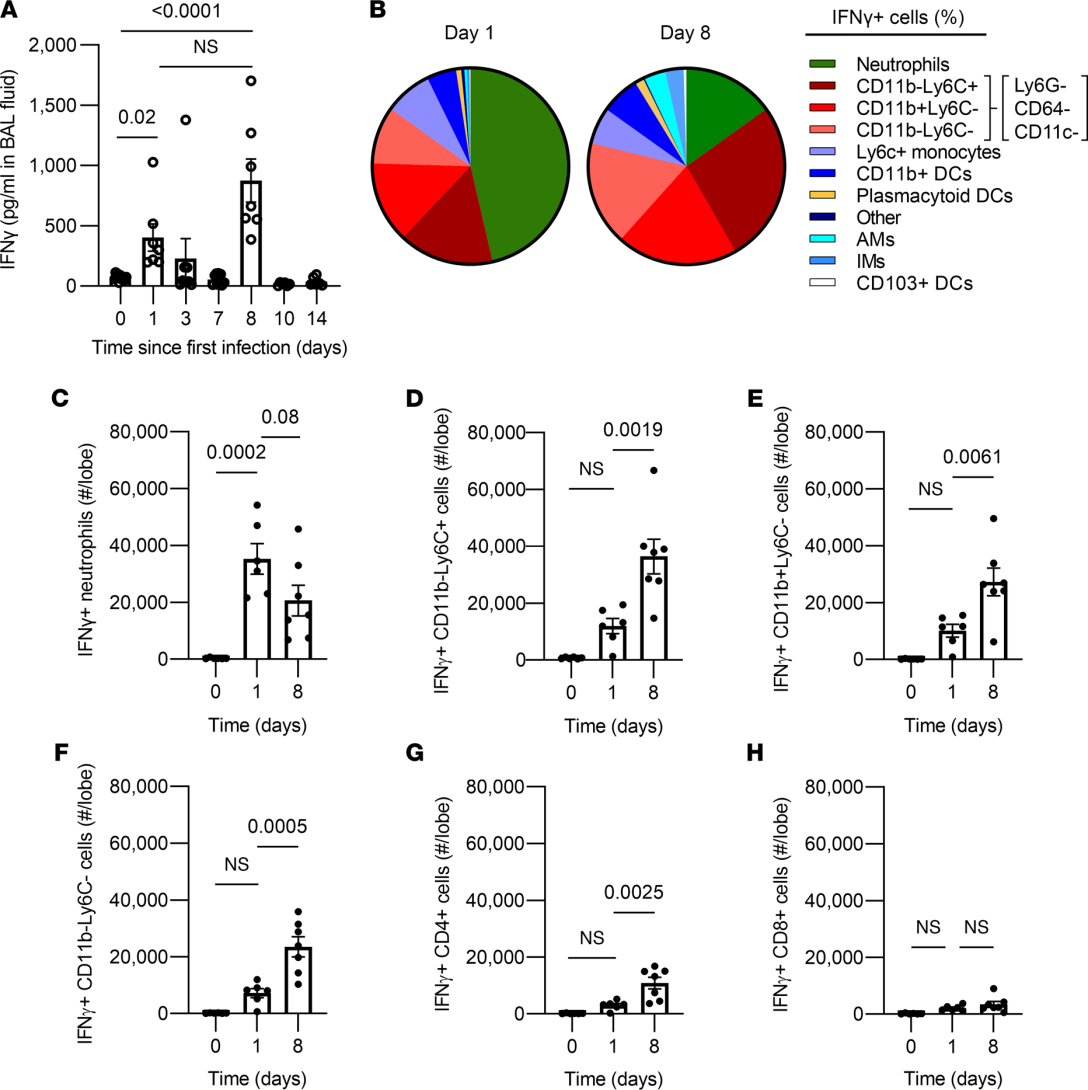
Cellular sources of IFN-γ during pneumococcal pneumonia. (**A**) ELISA measurement of IFN-γ concentration in broncho-alveolar lavage (BAL) fluids from left lungs at 0, 1, 3, and 7 days after the first Sp19F infection, as well as 1, 3, and 7 days after the second Sp19F infection that was delivered on day 7. Values are expressed as mean ± SEM. *n* = 6–11 mice per time point. One-way ANOVA with Tukey’s multiple-comparison test on log-transformed data was used to examine significance. (**B**–**H**) Intracellular cytokine staining was performed on single-cell suspensions from left lung digests from naive uninfected mice, 1 day after a single Sp19F infection (day 1), or 1 day after a second Sp19F infection (day 8), with *n* = 6–7 mice per time point. (**B**) Pie chart depicting cells that were IFN-γ^+^ expressed as an average of all IFN-γ^+^ cells in the lung. Myeloid cells were identified as described (21). (**C**) Numbers of IFN-γ^+^ neutrophils (identified as CD11b^+^Ly6G^+^). (**D**) Numbers of IFN-γ^+^ CD45^+^Ly6G^–^CD64^–^CD11c^–^CD11b^–^Ly6C^+^ cells. (**E**) Numbers of IFN-γ^+^ CD45^+^Ly6G^–^CD64^–^CD11c^–^CD11b^+^Ly6C^–^ cells. (**F**) Numbers of IFN-γ^+^ CD45^+^Ly6G^–^CD64^–^CD11c^–^CD11b^–^Ly6C^–^ cells. (**G**) Numbers of IFN-γ^+^ CD4^+^ cells (identified as CD19^–^CD8^–^CD4^+^ cells). (**H**) Number of IFN-γ^+^ CD8^+^ cells (identified as CD19^–^CD4^–^CD8^+^ cells). Values are expressed as mean ± SEM. One-way ANOVA Tukey’s multiple-comparison tests were used to examine significance.

During pneumococcal infections, neutrophils have been shown to produce significant amounts of IFN-γ within 1 day of infection (27, 28). Similarly, CD8^+^ effector T cells have been implicated in priming MHC-II upregulation on AM via IFN-γ after recovery from adenovirus infections (22). Because CD4^+^ T cells were dispensable, we considered whether other cells might be sources of IFN-γ during the initial infection stages. Reasoning that the IFN-γ to which AM respond would be in the airspaces where AM reside, we measured IFN-γ in bronchoalveolar lavage fluids collected during our infection model. IFN-γ concentrations were elevated 1 day after infection, declined, and then rose to even higher levels on day 8, 1 day after the second infection (Figure 3A). To determine the sources of IFN-γ at these time points, SCS from digested lung lobes were generated, and IFN-γ^+^ cells were discriminated using intracellular cytokine staining. This should include all local sources of IFN-γ, regardless of their intrapulmonary localization. After the first pneumococcal infection, many cells could express IFN-γ, but neutrophils were by far the most abundant sources in the lungs (Figure 3B). After the second infection that elicited higher amounts of IFN-γ, sources were even more diverse and heterogeneous, with neutrophils accounting for a smaller fraction and more prominent roles emerging for leukocyte subsets that were CD45^+^ and positive for either but not both of CD11b or Ly6c (Figure 3B). An additional subset of cells emerging as an IFN-γ source at day 8 failed to stain for any of the markers examined other than CD45 (Figure 3B). The total numbers of IFN-γ^+^ cells per lung peaked on day 1 for neutrophils but at day 8 for the other cell types (Figure 3, C–F). In separate analyses, CD4^+^ and CD8^+^ cells were examined, and the number of CD4^+^ cells but not CD8^+^ cells producing IFN-γ was significantly higher on day 8 than day 1, immediately after the second pneumococcal infection (Figure 3, G and H). These T cells would be components of the Ly6G^–^CD64^–^CD11c^–^CD11b^–^Ly6C^+^ sources of IFN-γ, with the remainder perhaps being NK cells and other innate lymphocytes. Together, these data highlight that there are many and diverse sources of IFN-γ during pneumococcal pneumonia. If IFN-γ is a driving factor for AM remodeling, then multiple different cells work together to provide this signal.

### IFN-γ is required for some but not all of the remodeled alveolar macrophage phenotype.

**Figure 4:**
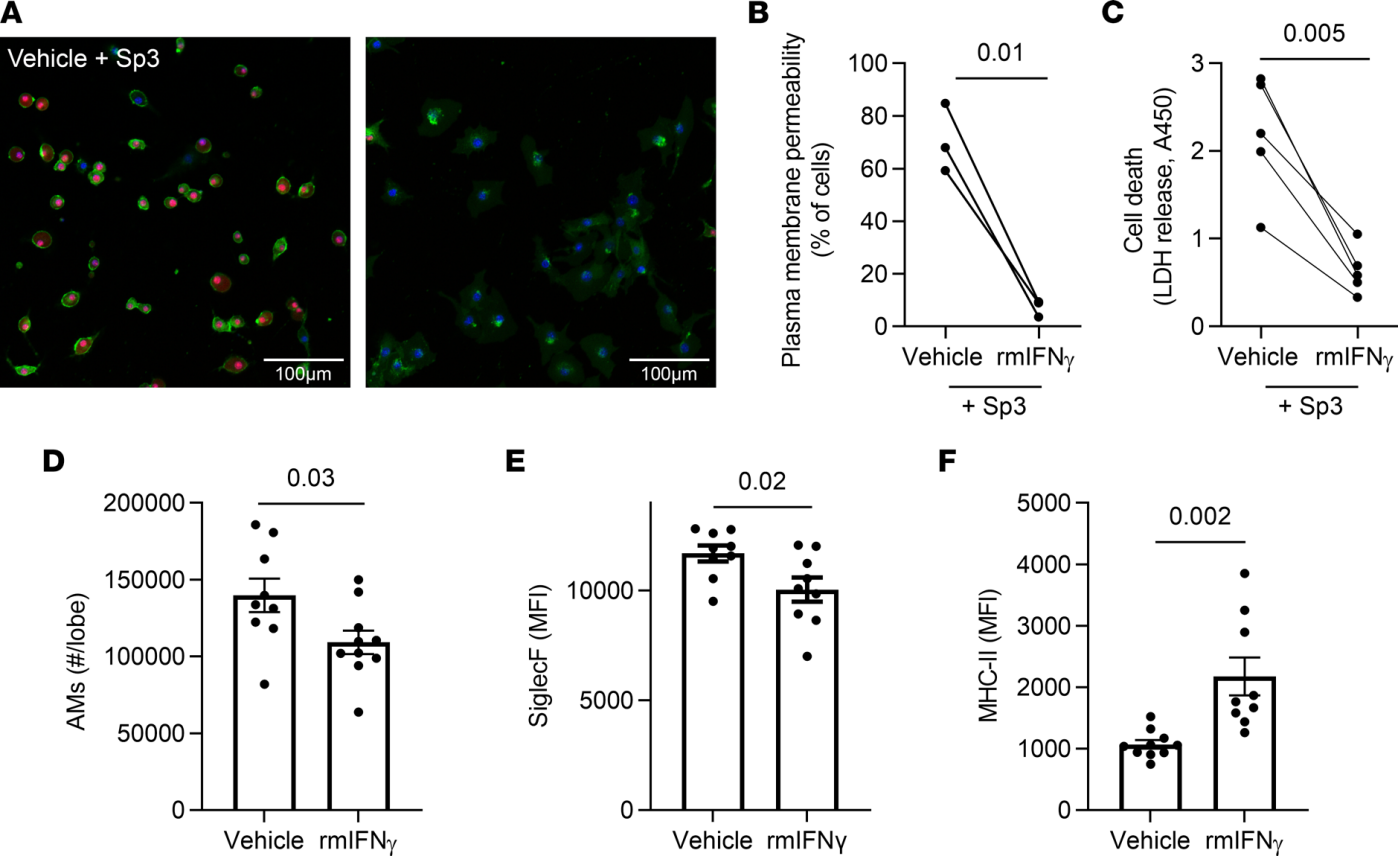
IFN-γ is sufficient to remodel and protect macrophages. (**A**–**C**) RAW264.7 cells were treated with recombinant mouse IFN-γ or vehicle prior to being infected with Sp3 for 2 hours. (**A**) Infected cells were stained with Hoechst (nucleus, blue), Cell Mask Green (plasma membrane, green) and Nuclear Red Dye 647 (permeable dye, red). Scale bar: 100 μm. (**B**) Quantification of cells with permeable plasma membranes, defined by nuclear red staining. *n* = 3 independent experiments. (**C**) Quantification of ell death, as measured by LDH in the supernatant. *n* = 5 independent experiments. Paired *t* test analyses were used to examine significance. (**D**–**F**) Seven- to 8-week-old mice were i.t. stimulated with IFN-γ or vehicle (1% BSA in saline) directed to the left lung twice at a 1-week interval, to mimic infection experiences. Four weeks after the second stimulation with IFN-γ, single-cell suspensions were created from left lung lobes and processed using flow cytometry. AM were identified as CD11c^+^SiglecF^+^CD64^+^ cells. *n* = 9–10 mice per group. (**D**) AM numbers were calculated from the frequency of total measured by FlowJo software. (**E** and **F**) Median fluorescent intensity (MFI) of Siglec F and MHC-II expression on AM. Values are expressed as mean ± SEM. Unpaired *t* test analyses were used to examine significance.

Since transcriptomes of AM in experienced lungs exhibit a signature of IFN-γ signaling (21), and since IFN-γ is necessary to remodel AMs after adenoviral infection (22), we investigated whether IFN-γ was sufficient to influence AM interactions with pneumococcus and the resulting remodeled phenotype. The immediate drop in AM numbers after infection (Figure 1B) is consistent with macrophage necroptosis induced by pneumococci (15), leading us to test whether IFN-γ could affect macrophage survival. We used the RAW264.7 macrophage-like cell line, which accurately reports pneumococcus-induced necroptosis (15), to investigate this concept. When stimulated with IFN-γ, these cells became more resilient and better able to survive pneumococcal infection (Figure 4, A–C). Thus, macrophage numbers may be influenced by this cytokine. To test whether IFN-γ was sufficient to influence the surface marker phenotype of AM, uninfected mice received i.t. instillations of either IFN-γ or vehicle twice with a 1-week interval (to mimic the timing of infections that establish immune protection) and the AM were immunophenotyped 4 weeks later (to model the wait period in experienced lung studies). Mice that received prior IFN-γ stimulation exhibited slightly but significantly fewer AM per lung compared with those stimulated with vehicle (Figure 4D), suggesting that IFN-γ has complex and context-specific effects on macrophage survival. Examining their surface marker phenotypes, mice stimulated with IFN-γ had AM with significantly less Siglec F and significantly more MHC-II on their surface, compared with control mice stimulated with vehicle (Figure 4, E and F). Although less pronounced, these results mirror the remodeling of AM observed in lungs with pneumococcal pneumonia experience (Figure 1) (21). Thus, IFN-γ is sufficient to push AM phenotypes toward the surface marker changes observed in pneumococcus-experienced mice.

**Figure 5:**
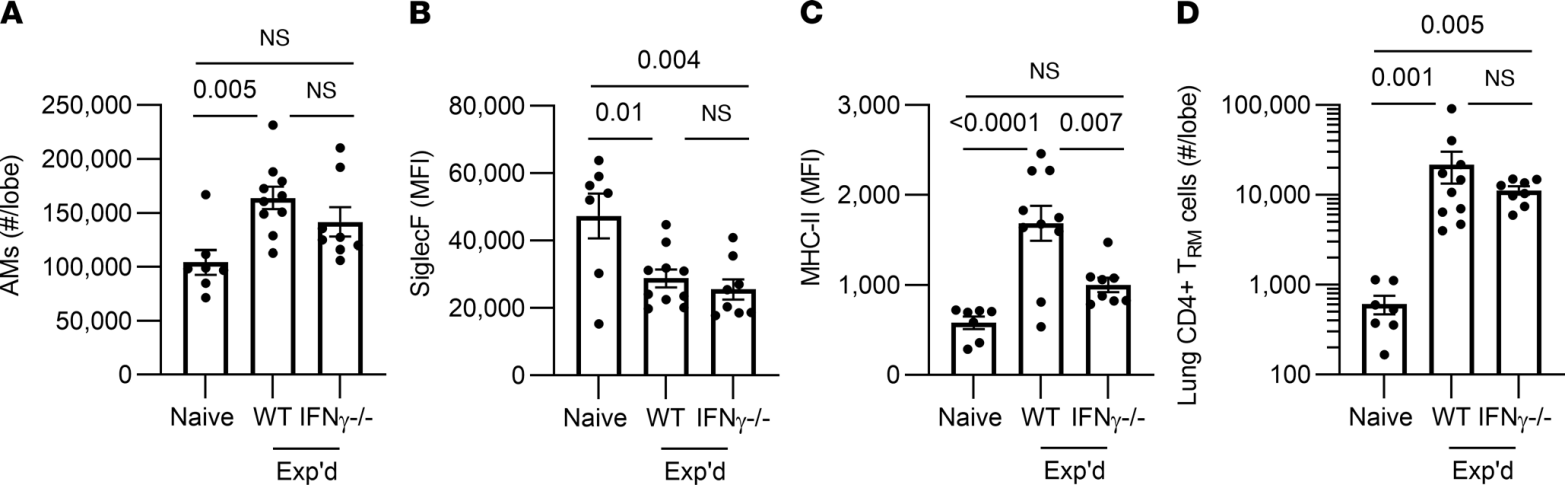
Roles for IFN-γ in remodeling of AM. Wild type (WT) C57BL/6 or IFN-γ^–/–^ mice were i.t. challenged with Sp19F or saline directed to the left lung twice, referred to as experienced (Exp’d) or Naive, respectively. After 4–6 weeks (to allow resolution of inflammation), the left lung lobes of these mice were immunophenotyped. *n* = 7–10 mice per group. Values are expressed as mean ± SEM. AM were identified as CD11c^+^SiglecF^+^CD64^+^ cells. Numbers were calculated from the frequency of total measured by FlowJo software. Median fluorescent intensity (MFI) of Siglec F and of MHC-II were calculated on AM from each mouse. One-way ANOVA with Tukey’s multiple-comparison test was used to examine significance. (**A**–**D**) Lung CD4^+^ resident memory T (T_RM_) cells were identified as extravascular CD4^+^CD69^+^CD11a^+^CD44^+^CD62L^–^ live cells, and numbers were calculated from the frequency of total gated events measured by FlowJo software. Kruskal-Wallis with Dunn’s multiple-comparison test was used to examine significance.

To complement IFN-γ gain of function, we tested whether IFN-γ loss of function would prevent AM from gaining some or all of the experienced phenotype. Wild type (WT) and IFN-γ^–/–^ mice were ushered through the pneumococcal experience model and immunophenotyped 4 week after the last infection. Numbers of AM per experienced lung did not differ in IFN-γ^–/–^ mice compared with WT mice (Figure 5A). The decrease in Siglec F expression was observed in AM of experienced IFN-γ^–/–^mice, with no significant difference from WT mice (Figure 5B). Thus, this aspect of immune remodeling did not require IFN-γ. In contrast, surface MHC-II of AM from experienced IFN-γ^–/–^ mice was significantly less than in WT counterparts and was not different from naive mice without a pneumococcal infection history (Figure 5C), indicating a requirement for IFN-γ for this outcome. Since CD4^+^ T_RM_ cells are another critical component of immunity in lungs with pneumococcal experience (23–25), we investigated whether loss of IFN-γ would impact numbers of these cells. Like AM Siglec F but contrasting with AM MHC-II, the CD4^+^ T_RM_ cell numbers were unaffected by deficiency of IFN-γ (Figure 5D). Thus, IFN-γ has essential but select roles in remodeling lung immunity after recovery from pneumococcal pneumonia — in particular, being responsible for the increased MHC-II on AM.

### IFN-γ signals directly to alveolar macrophages.

**Figure 6:**
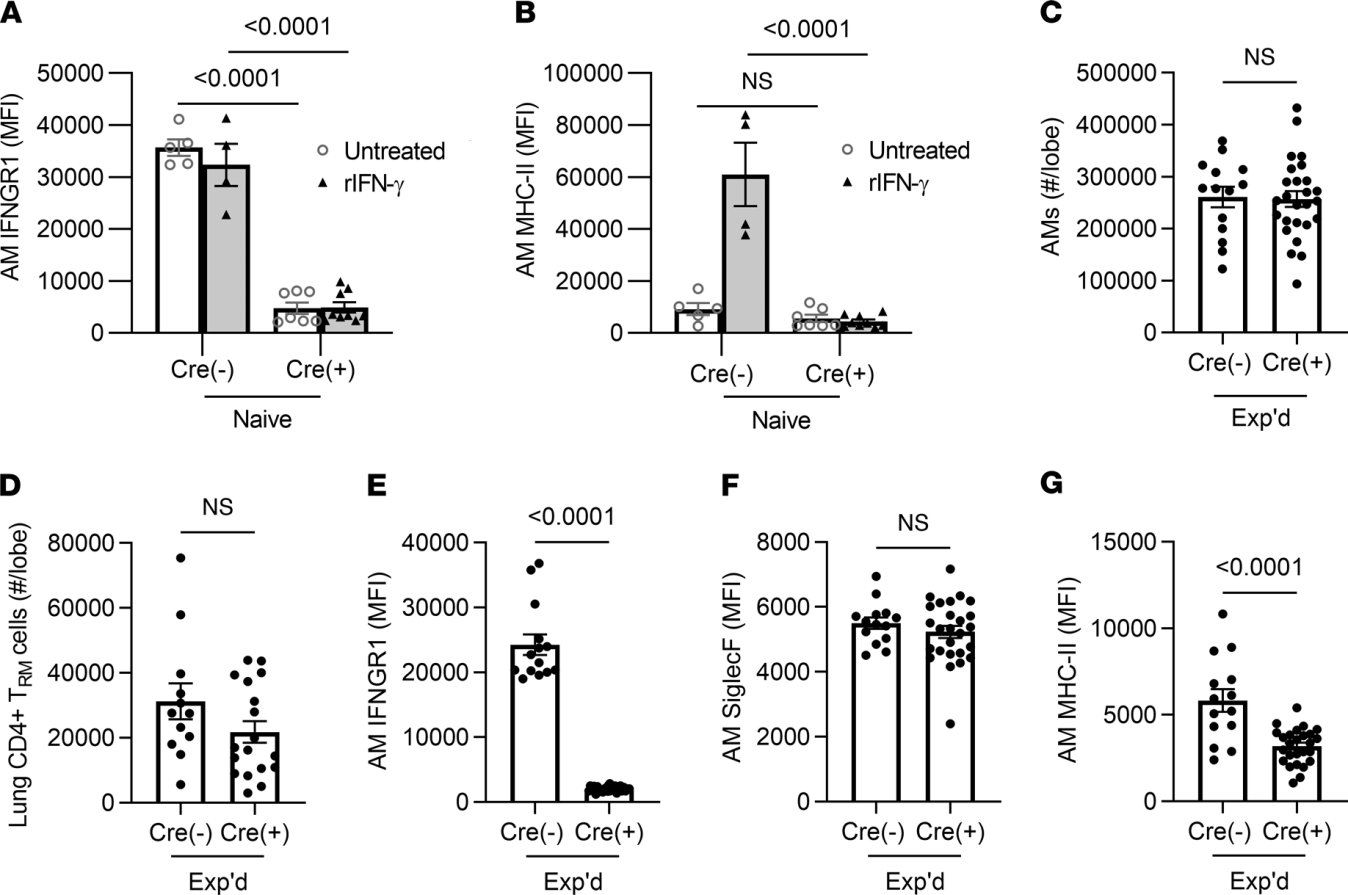
Roles for the IFN-γ receptor on AM in their remodeling after pneumococcus infections. CD11c-Cre and IFNGR1-floxed mice were crossed to produce IFNGR1 mutation in CD11c^+^ cells. (**A** and **B**) Acute responses to IFN-γ. MFI of MHC-II and IFNGR1 were measured on AM, identified as live CD11c^+^SiglecF^+^CD64^+^ cells in single-cell suspensions from left lung lobes. Cre^–^ and Cre^+^ mice were instilled i.n. with 200 ng/mL IFN-γ and compared with uninstilled controls. Values are expressed as mean ± SEM. *n* = 4–9 mice/group. Two-way ANOVA followed by Tukey’s multiple comparison test was used to calculate significance. (**C**–**G**) AM and T cells in the left lung lobes of mice with prior pneumococcal experience. (**C**) Enumeration of AM, identified as live CD11c^+^SiglecF^+^CD64^+^ cells. *n* = 14–26 mice per group. Values are expressed as mean ± SEM. Unpaired *t* tests were used to examine significance. (**D**) Enumeration of lung CD4^+^ resident memory T (T_RM_) cells, identified as extravascular CD4^+^CD69^+^CD11a^+^CD44^+^CD62L- cells, with numbers calculated from the frequency of total measured by FlowJo software in experienced lungs from Cre^–^ and Cre^+^ mice. *n* = 12–18 mice/group. Values are expressed as mean ± SEM. Unpaired *t* tests were used to examine significance. (**E**–**G**) Median fluorescent intensity (MFI) of IFNGR1, Siglec F, and MHC-II, measured on AM in single-cell suspensions from left lung lobes. AM were identified as CD11c^+^SiglecF^+^CD64^+^ cells. *n* = 14–26 mice/group. Values are expressed as mean ± SEM. Unpaired *t* tests were used to examine significance.

To test whether the essential role for IFN-γ in remodeling AM after pneumococcal pneumonia required IFN-γ signaling to AM, we crossed *Ifngr1*-floxed mice with *Itgax*-driven Cre transgenic mice to delete IFN-γ receptors from CD11c^+^ cells (29, 30). As designed, the IFN-γ receptor was effectively targeted, with diminished expression of the receptor on AM from Cre^+^ mice compared with their Cre^–^ littermates (Figure 6A). The instillation of IFN-γ to the lungs was sufficient to increase MHC-II on AM, but this was abrogated in the Cre^+^ mice without IFN-γ receptors on these cells (Figure 6B). Littermates that were Cre^+^ and Cre^–^ were ushered through the pneumococcal experience model and immunophenotyped 4 weeks after the last Sp19F challenge. AM were present in similar numbers in the experienced Cre^–^ and Cre^+^ mice (Figure 6C). The AM loss of IFN-γ receptor did not affect the deposition of CD4^+^ T_RM_ cell numbers in the experienced lung (Figure 6D). AM retained their differences in IFN-γ receptor expression (Figure 6E). Exactly analogous to the results with IFN-γ^–/–^ mice, the experienced Cre^+^ mice exhibited no changes in Siglec F levels but significantly less MHC-II compared with experienced Cre^–^ controls (Figure 6, F and G), suggesting that this element of immune remodeling depends on IFN-γ signaling to AM directly. These data reveal that the stable and prolonged increase in surface MHC-II is dependent on IFN-γ signaling directly to AM.

**Figure 7:**
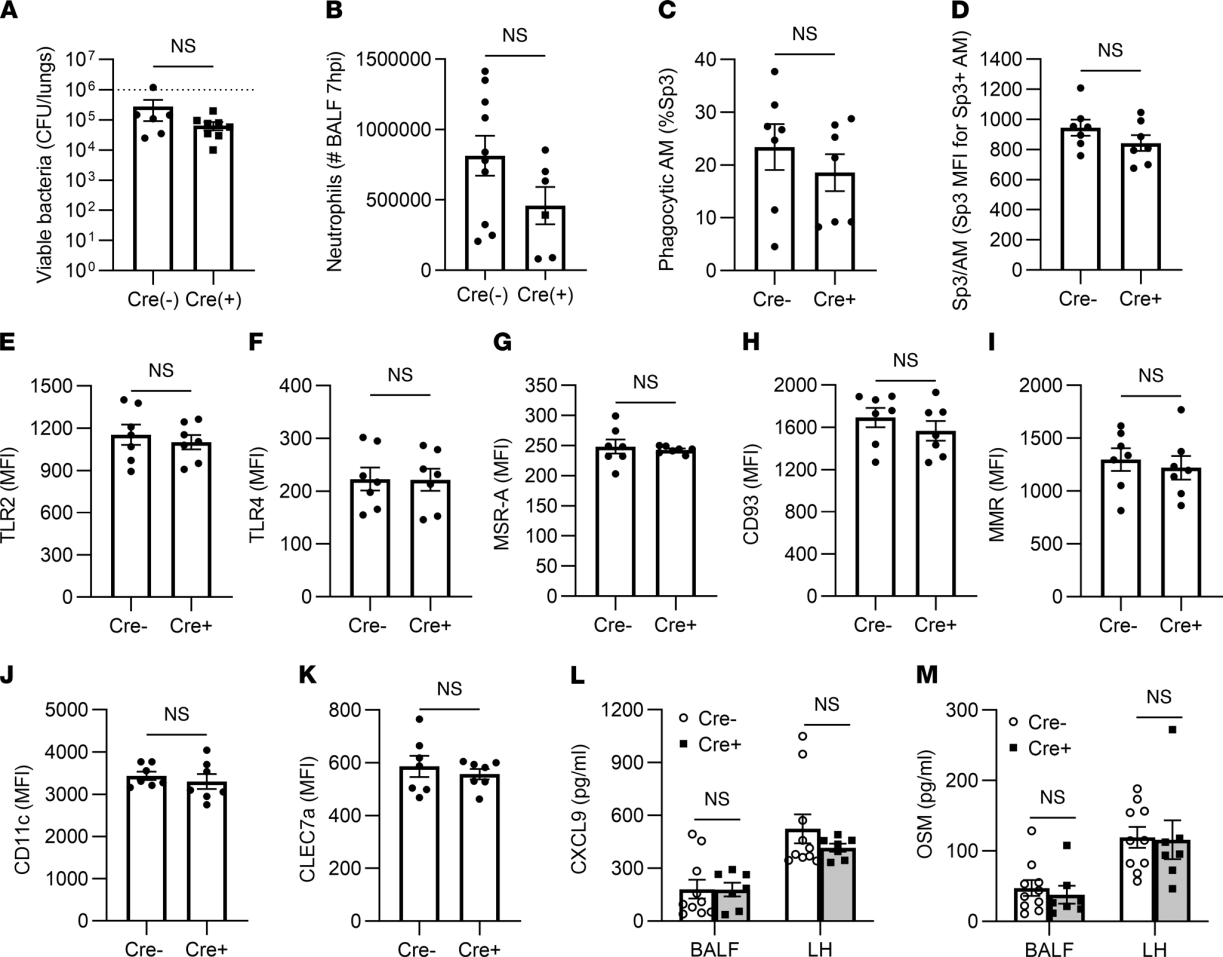
Immune activities in the absence of IFN-γ receptor on AM. CD11c-Cre and IFNGR1-floxed mice were crossed to produce IFNGR1 mutation in CD11c^+^ cells. Prior pneumococcal experience was generated as described in Methods for all mice in the studies shown. AM and neutrophils were identified as CD11c^+^SiglecF^+^ and Ly6G^bright^ cells in bronchoalveolar lavage fluids. (**A**) Viable bacteria per lung, after mice were infected i.t. 24 hours previously with Sp3. *n* = 6–8 mice per group. (**B**) Neutrophils in the air spaces of mice with 7 hours of Sp3 pneumonia, quantified in BAL fluids. *n* = 6–10 mice/group. (**C**) Fraction of AM that were actively phagocytic, expressed as the percentage of total AM that were associated with PKH67-labeled bacteria 40 minutes after instillation. *n* = 7 mice per group. (**D**) Amount of Sp3 phagocytized by AM that were actively phagocytic, expressed as the MFI of those AM that were PKH67^+^ 40 minutes after instillation of fluorescent bacteria. (**E**–**K**) Surface levels of phagocytic receptors and pattern-recognition receptors on AM from the phagocytosis experiment, expressed as MFI. (**L** and **M**) Concentrations of CXCL9 and OSM in BAL fluids and lung homogenates collected 7 hours after Sp3 infection. *n* = 7–10 mice/group. For **A**–**K**, data were compared using unpaired *t* tests. For **L** and **M**, data were compared using 2-way ANOVA with Tukey’s multiple-comparison test. Values are expressed as mean ± SEM.

The effects of IFN-γ interruption on AM MHC-II prompted examination of whether and which responses to infection in experienced lungs might depend on IFN-γ signaling to AM. Integrated immune activities including net antibacterial defense (Figure 7A) and early neutrophil recruitment (Figure 7B), both of which are stimulated by heterotypic immunity (21, 23, 24), were unaffected by the loss of the IFN-γ receptors on AM. To examine AM phagocytosis in these experienced lungs, mice were challenged with fluorescently labeled serotype-mismatched pneumococci in the air spaces. Within 40 minutes, 5%–40% of the AM contained labeled bacteria, with no significant differences due to loss of the IFN-γ receptor (Figure 7C). The relative bacterial content per cell among AM that were phagocytic was not affected by genotype (Figure 7D). The surface levels of relevant phagocytic receptors and pattern-recognition receptors were examined, and none were impacted by the loss of IFN-γ receptor on Cre^+^ AM (Figure 7, E–K). Remodeled AM exhibit increased expression and pulmonary content of the chemokine CXCL9 and the cytokine OSM (21). The interruption of IFN-γ signaling did not affect these outcomes, whether measured in the airspaces or in lung tissues (Figure 7, L and M). Taken together, these data demonstrate that loss of IFN-γ signaling on CD11c^+^ cells has only a very select effect on AM remodeling and immunity against pneumococcus in experienced lungs, enhancing surface expression of MHC-II but not any of the other variables measured.

### Alveolar macrophages in recovered lungs do not depend on CCR2.

**Figure 8:**
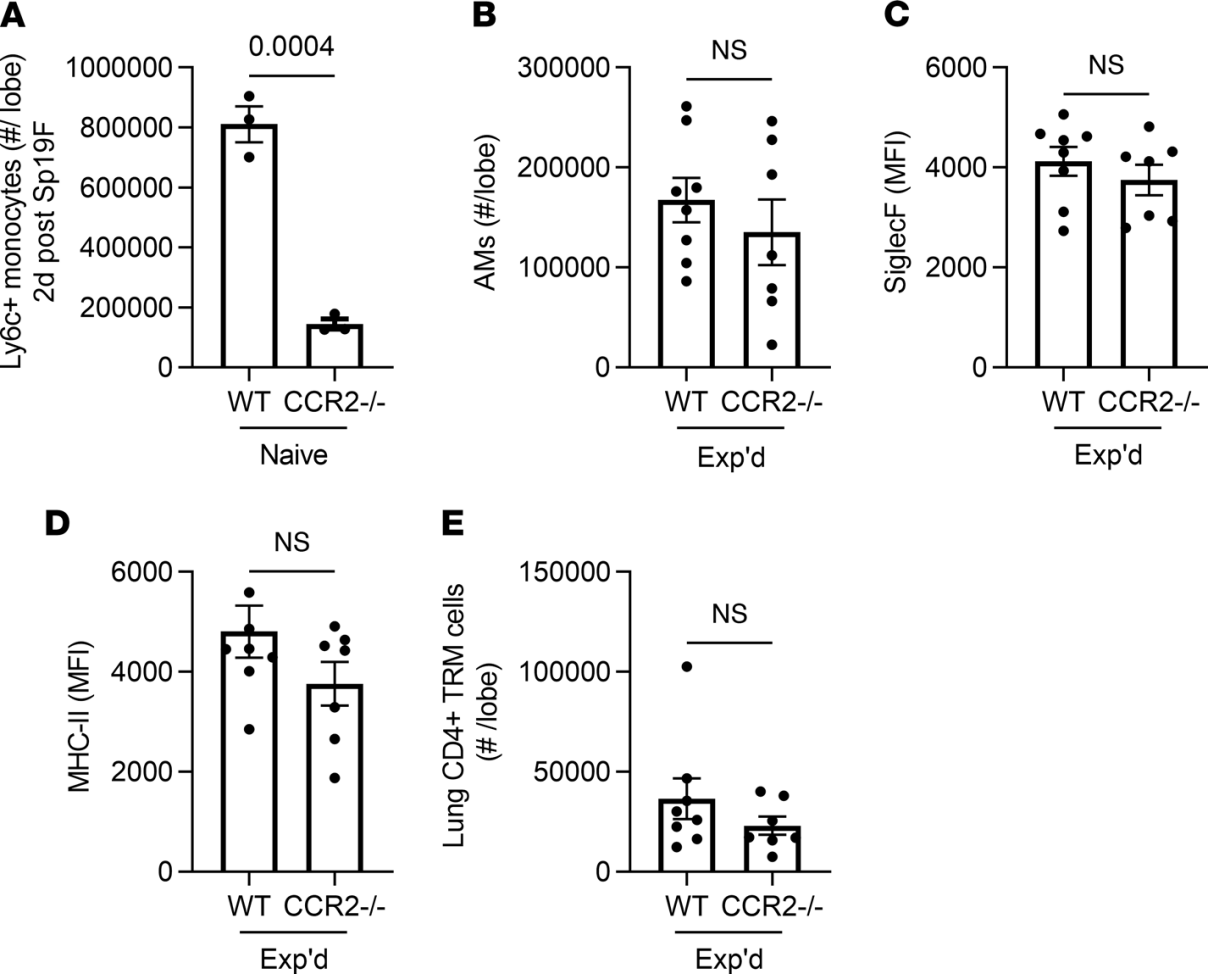
CCR2 and the experienced AM phenotype. (**A**) Enumeration of extravascular monocytes in single-cell suspensions from lung lobes of WT or CCR2^–/–^ mice infected with Sp19F for 2 days, defined as live CD11c^–^SiglecF^–^CD11b^+^MHCII^–^Ly6C^+^ cells using a similar schema to the one detailed in Figure 1A. *n* = 3 mice per group. (**B**) AM were identified as live CD11c^+^SiglecF^+^CD64^+^ cells using a similar schema to the one detailed in Figure 1A, and numbers were calculated from the frequency of total measured by FlowJo software. (**C** and **D**) Median fluorescent intensity (MFI) of Siglec F and MHC-II expression on AM. (**E**) Lung CD4^+^ resident memory T (T_RM_) cells were identified as extravascular CD4^+^CD69^+^CD11a^+^CD44^+^CD62L^–^ cells, and numbers were calculated from the frequency of total measured by FlowJo software. For **B**–**E**, *n* = 8–10 mice per group. Throughout, values are expressed as mean ± SEM, and unpaired *t* tests were used to examine significance.

IFN-γ is essential for remodeling AM after adenoviral infections in which there is no replacement of AM by recruited monocytes (22), and the above data suggest that IFN-γ contributes to select aspects of AM remodeling after pneumococcal pneumonia. In contrast to adenovirus, lungs recovered from influenza infection experienced replacement of the initial AM by recruited monocyte-derived AM (14). After recovery from pneumococcus, transcriptomes of the remodeled AM pool also suggest the possibility of AM of monocyte origin (21). Monocyte recruitment into the inflamed lungs often depends upon the chemokine receptor CCR2 (31–34). Early monocyte recruitment elicited by acute pneumococcus infection of the lungs depends on CCR2 (Figure 8A). To determine whether CCR2-mediated monocyte recruitment was essential to the remodeled AM phenotype, we examined WT and CCR2^–/–^ mice that recovered from prior pneumococcal pneumonias. The CCR2^–/–^ mice demonstrated no differences from WT mice related to AM numbers (Figure 8B) or surface levels of Siglec F (Figure 8C) or MHC-II (Figure 8D). Although the loss of CCR2 impairs CD8^+^ T_RM_ cell accumulation in influenza models (35), CD4^+^ T_RM_ cell accumulation after pneumococcal pneumonia did not depend on this chemokine receptor (Figure 8E). These data reveal that CCR2 mediates acute monocyte recruitment during pneumococcal pneumonia, but CCR2-mediated monocyte recruitment is not essential for AM numbers or the changes in AM surface marker phenotype in lungs that have recovered from pneumococcal pneumonia.

### Alveolar macrophages of distinct origins remodel similarly after pneumonia recovery.

**Figure 9:**
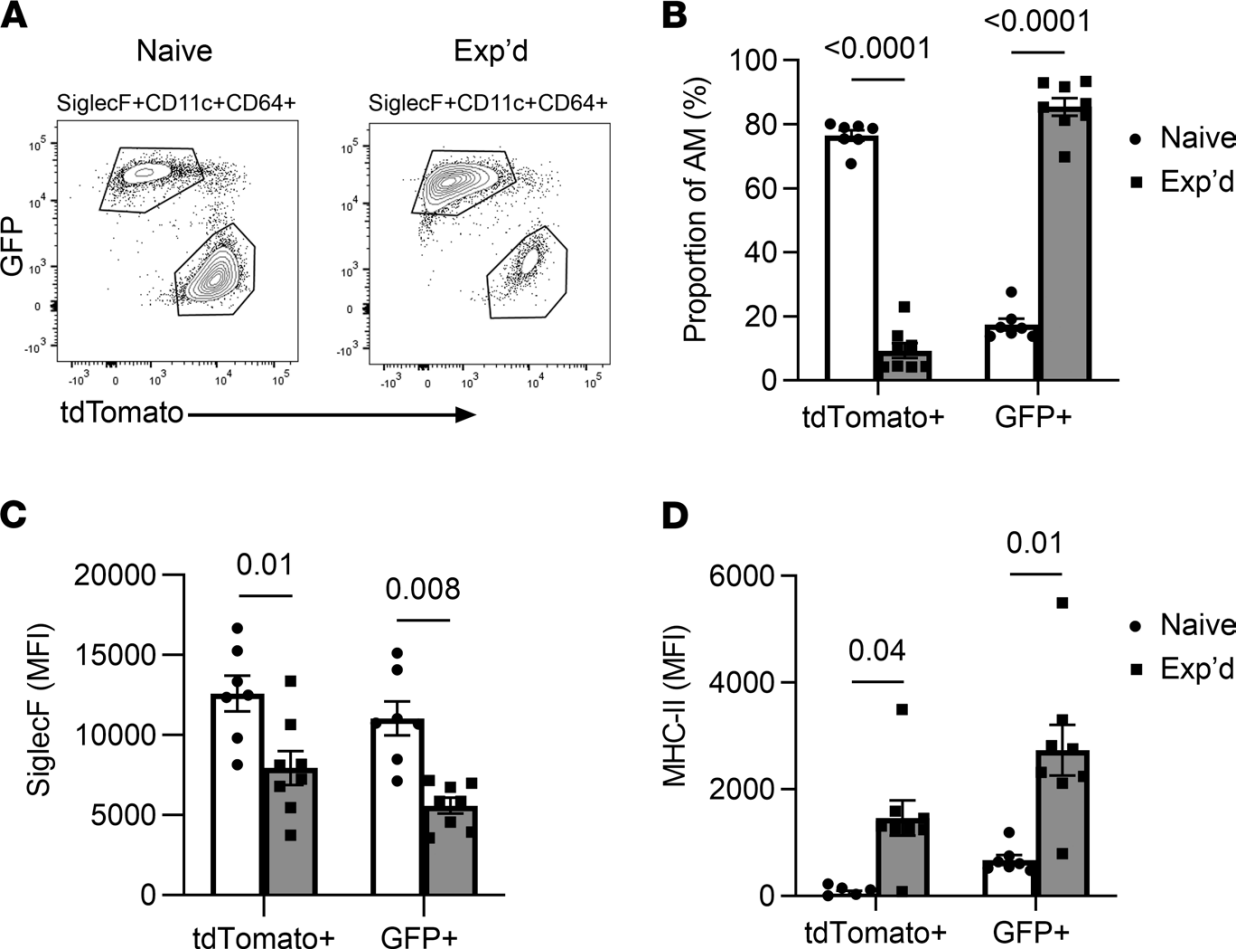
AM from different origins remodel similarly after pneumococcal infections. A lineage-tracing mouse model allowed the differentiation of AM of embryonic origin (TdTomato^+^) from those that derived from adult hematopoietic stem cells (GFP^+^). Naive and experienced mice had prior exposures to saline or pneumococcus Sp19F in their lungs, respectively. (**A**) Both naive and experienced lungs contain distinct populations of TdTomato^+^ and GFP^+^ AM. (**B**) Percentages of AM subsets in naive and experienced lungs. (**C**) Median fluorescent intensity (MFI) of Siglec F expression on AM subsets in naive and experienced lungs. (**D**) MFI of MHC-II expression on AM subsets in naive and experienced lungs. For all, *n* = 7–8 mice per group, values are expressed as mean ± SEM, and unpaired *t* tests were used to examine the effect of experience on AM subsets.

To definitively determine whether AM remodeling in lungs with prior pneumococcal experience involves monocyte recruitment, we used lineage tracing to differentiate AM that derived from embryonic versus adult hematopoietic stem cell (HSC) origin (10, 36–38). With this mouse model, the embryonic-origin macrophages express the red fluorescent tdTomato protein, whereas the adult monocyte-derived macrophages express green fluorescent protein (GFP) instead (10, 36–38). In naive mice, most but not all AM were tdTomato^+^, indicating that they were predominantly from embryonic origin (Figure 9, A and B). In contrast, for mice with prior experience with pneumococcal pneumonias, most but not all AM were GFP^+^, indicating that the majority were derived from adult HSC origin (Figure 9, A and B). These data confirm that AM in the remodeled pool of pneumococcus-experienced lungs include more AM that are differentiated from recruited monocytes (21). To determine whether one or the other or both lineages remodeled their surface phenotypes after resolution from pneumonia, we compared Siglec F and MHC-II on each subset of cells. Both AM subsets exhibited a significant decrease in Siglec F in experienced lungs compared with their naive counterparts (Figure 9C). Both AM subsets exhibited a significant increase in MHC-II in experienced lungs compared with their naive counterparts (Figure 9D). Altogether, these data reveal that phenotypic remodeling after pneumococcal experience is not confined to either the original AM or the AM products from new cells recruited into the lung; rather, both AM subsets are similarly subject to remodeling. Although monocyte-derived AM are major components of the remodeled AM pool after recovery from pneumococcal pneumonia, the AM of embryonic origin and of monocyte origin display matching phenotypic characteristics. These results suggest that the tissue microenvironment exerts a dominant influence on AM phenotype, regardless of developmental origin.

### Although characteristics are shared, alveolar macrophages of distinct origins are not equivalent.

**Figure 10:**
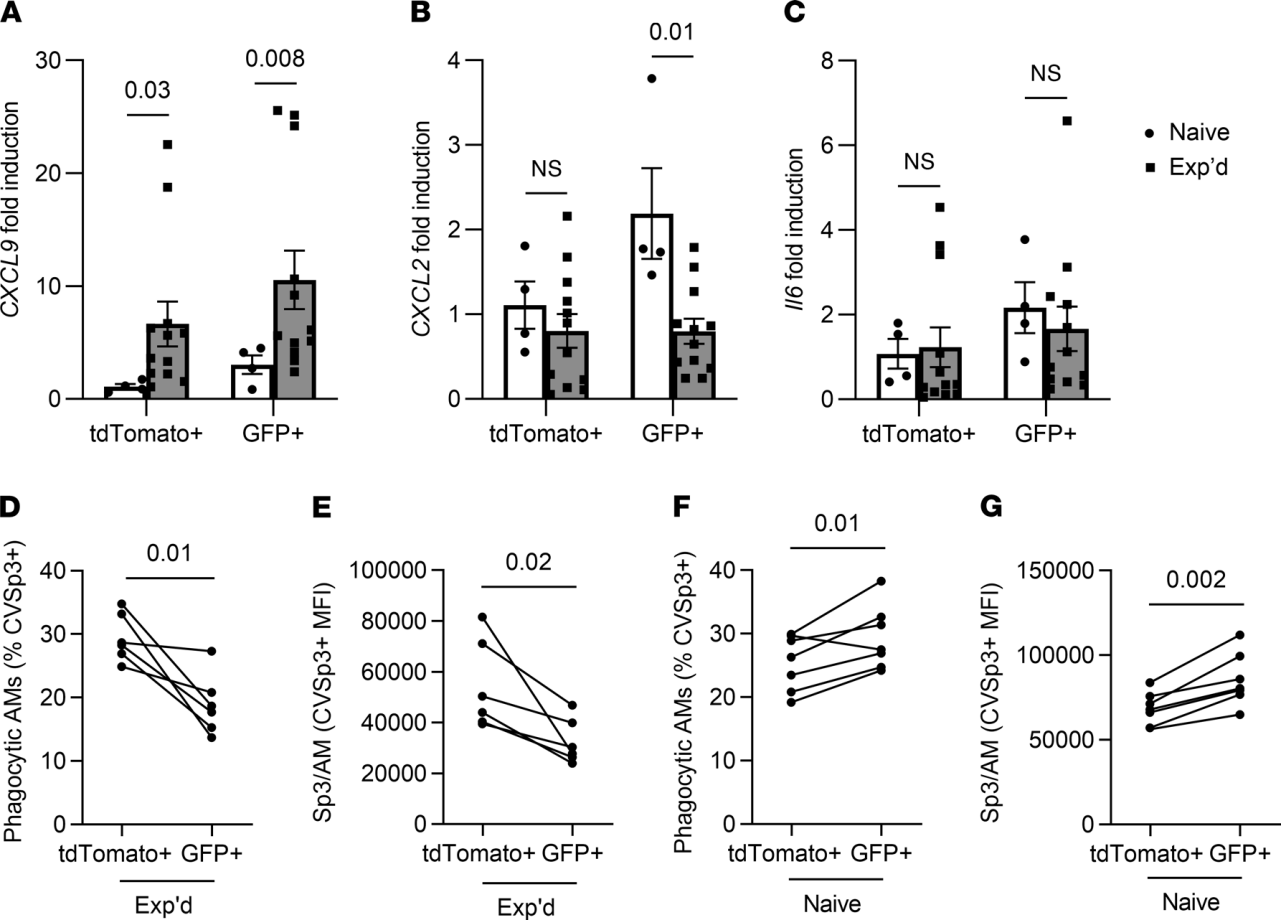
Recent recruits differ functionally from the AM that were initially resident in the experienced lung. A lineage-tracing mouse model allowed the differentiation of AM of embryonic origin (TdTomato^+^) from those that derived from adult hematopoietic stem cells (GFP^+^). (**A**–**G**) Naive and experienced mice had prior exposures to saline or pneumococcus Sp19F in their lungs, respectively, before being challenged with a mismatched Sp3 serotype prior to AM collection for studies of cytokine expression (**A**–**C**) or phagocytosis (**D**–**G**). (**A**–**C**) Fold induction of *Cxcl9* (**A**), *Cxcl2* (**B**), and *Il*6 (**C**) transcripts measured using quantitative PCR of sorted tdTomato^+^ and GFP^+^ AM, normalized to average induction in naive tdTomato^+^ AM for each gene. *n* = 4–12 mice per group. Values are expressed as mean ± SEM, and unpaired Mann-Whitney *U* tests were used to examine the effect of experience on each AM subset. (**D**) Fraction of AM of distinct origin that were actively phagocytic within experienced lungs, expressed as the percentage of total AM of that origin (TdTomato^+^ or GFP^+^) that were associated with ClaretVue-labeled bacteria 40 minutes after instillation. (**E**) Amount of Sp3 phagocytized by AM that were actively phagocytic within experienced lungs, expressed as the MFI of those AM that were ClaretVue^+^. (**D** and **E**) *n* = 6 mice per group, with lines connecting AM subsets of distinct origin within a given mouse lung. (**F**) Fraction of AM of distinct origin that were actively phagocytic within naive lungs, expressed as the percentage of total AM of that origin (TdTomoato^+^ or GFP^+^) that were associated with ClaretVue-labeled bacteria 40 minutes after instillation. (**G**) Amount of Sp3 phagocytized by AM that were actively phagocytic within naive lungs, expressed as the MFI of those AM that were ClaretVue^+^. (**F** and **G**) *n* = 7 mice per group, with lines connecting AM subsets of distinct origin within a given mouse lung. The experienced mice in **D** and **E** and naive mice in **F** and **G** were collected in different experiments; therefore, data should not be compared across the former and latter panels. For **D**–**G**, paired *t* tests were used to examine significance.

In addition to new resting phenotypes that include lesser Siglec F and greater MHC-II, remodeled AMs have different responses to challenge‚ such as increases in CXCL9 after pneumococcus-induced remodeling, in CXCL2 after adenovirus-induced remodeling or in IL-6 after influenza-induced remodeling (14, 21, 22). We tested whether there were distinctions between expression of these cytokines in AM of embryonic origin versus newer recruits in experienced lungs by measuring transcripts in sorted cells using quantitative PCR. Consistent with prior studies of remodeling after pneumococcal pneumonia, the AM from experienced lungs had increased *Cxcl9* induction during infection compared with AM from infected lungs of naive mice (Figure 10A). *Cxcl9* expression was similar between tdTomato^+^ embryonic origin AM and GFP^+^ monocyte-derived AM (Figure 10A), further confirming that the local tissue microenvironment influences responses of these cells in experienced lungs more than their ontogeny does. Unlike after adenovirus or influenza infections (35, 39), AM from lungs that have recovered from pneumococcal pneumonia do not demonstrate elevated *Cxcl2* or *Il6* expression, whether of embryonic or adult HSC origin (Figure 10, B and C). Altogether, these data reveal that the remodeled AM biology in lungs recovering from pneumococcal pneumonia differs from that reported for other infections. For surface marker and cytokine phenotypes resulting from remodeling, the initial AM and the more recently recruited AM become similarly altered.

The defining feature of AM is their ability to phagocytize substances in the air spaces (40), so we examined whether these different AM subsets differed at ingesting pneumococcus in experienced lungs. Mismatched serotypes precluded opsonophagocytosis driven by antibodies against capsular polysaccharides. To test whether embryonically derived and adult HSC–derived AM had different phagocytic capacity, the lineage-tracing mice were ushered through the pneumococcal experience protocol and challenged after 4 weeks with fluorescently labeled serotype-mismatched pneumococci in the air spaces. Within 40 minutes, 25%–35% of the tdTomato^+^ AM were positive for the labeled bacteria, but significantly smaller fractions of the GFP^+^ AM from the same lungs were demonstrably phagocytic (Figure 10D). Comparing relative bacterial content per AM among cells that were phagocytic, greater fluorescent bacteria median fluorescent intensity (MFI) in the tdTomoto^+^ AM compared with their GFP^+^ counterparts from the same lungs again suggested that the embryonic origin AM were more phagocytic (Figure 10E). To test whether this observed phagocytic phenotype was due to pneumococcal experience, naive mice were challenged with fluorescently labeled pneumococci. In contrast to experienced mice, tdTomato^+^ AM from naive lungs were significantly less labeled (Figure 10F) and exhibited less fluorescent bacteria intensity (Figure 10G) compared with their GFP^+^ AM counterparts. These results reveal that the AM of embryonic or adult origin manifest functional differences, and these functions are influenced by infection history.

**Figure 11:**
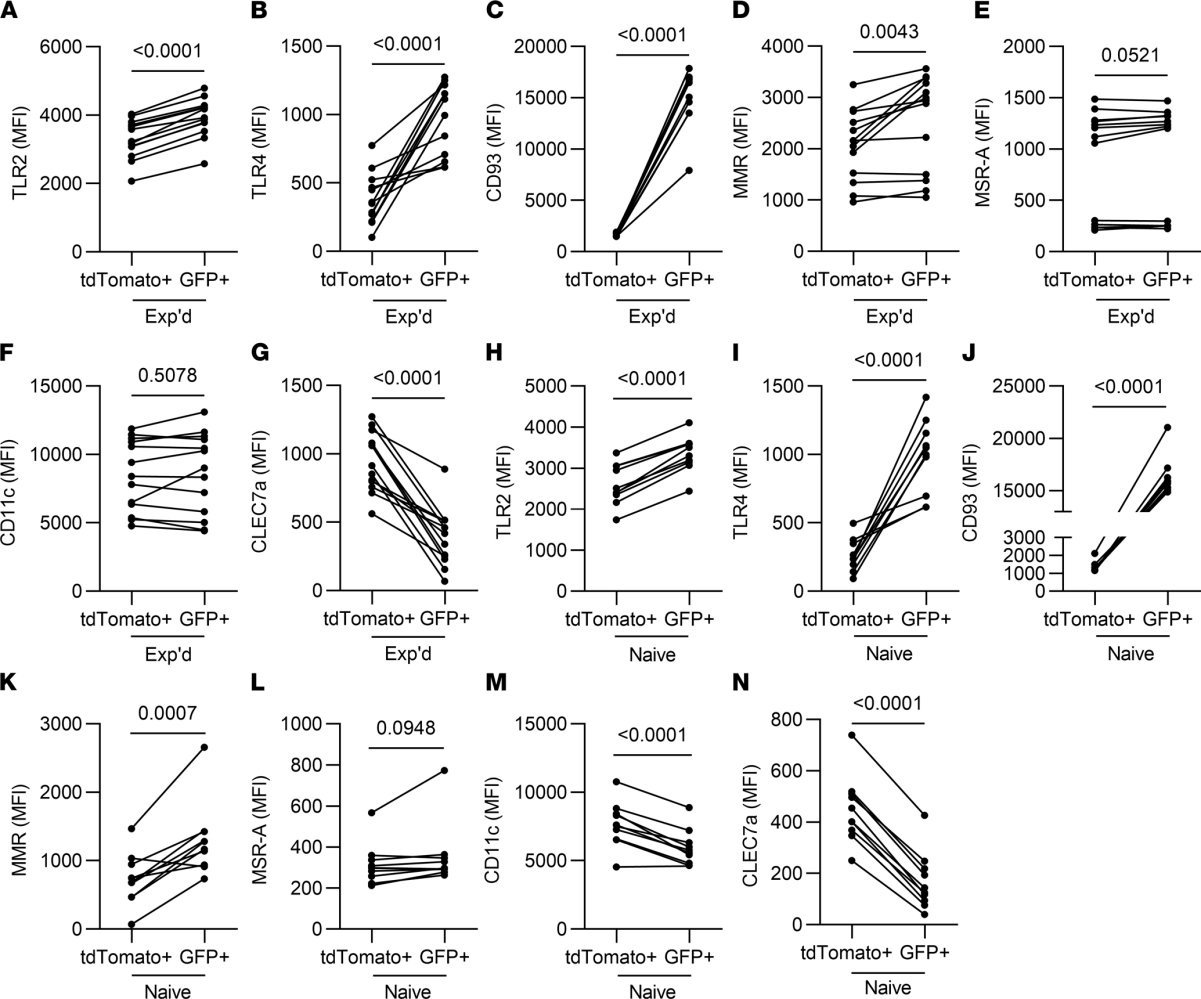
Differential surface levels of phagocytic receptors and pattern-recognition receptors on AM of distinct origins, in naive and experienced lungs. A lineage-tracing mouse model allowed the differentiation of AM of embryonic origin (TdTomato^+^) from those that derived from adult hematopoietic stem cells (GFP^+^). (**A**–**G**) Experienced mice were ushered through the pneumococcal experience model, (Experienced, Exp’d). (**H**–**N**) Naive mice were left unchallenged (Naive). AM were identified as live CD11c^+^SiglecF^+^CD64^+^ cells in single-cell suspensions from the left lung lobe. Each panel shows median fluorescent intensity (MFI) of the indicated receptor on the indicated AM subsets, with lines connecting AM subsets of distinct origin within a given mouse lung. *n* = 7–11 mice per group. Paired *t* test analysis was used to examine significance.

The distinct phagocytic activities of AM from different origins suggested the possibility that phagocytic receptors and pattern-recognition receptors might differ between these AM subsets. The lineage tracing mice were leveraged to compare AM from embryonic and adult HSC origin in experienced and naive mice (Figure 11). Compared with AM of embryonic origin, the more recently recruited AM showed very pronounced increases in CD93, regardless of experience; modest increases in TLR2, TLR4, and the mannose receptor, regardless of experience; no differences in scavenger receptor A, regardless of experience; a modest decrease in CD11c that was observed in naive but not experienced lungs; and a decrease in dectin-1, regardless of experience (Figure 11). These results reveal that AM of disparate origins exhibit extensive differences in expression of phagocytic receptors and pattern-recognition receptors. Differences between AM subsets in these phagocytic receptors and pattern-recognition receptors were observed in both naive and experienced lungs.

## Discussion

After recovery from pneumococcal pneumonia, AM become phenotypically, transcriptionally, and metabolically altered (21). In this report, we found that the AM pool was highly dynamic after pneumococcal infection, with loss within the initial AM pool and recruitment of monocytes that became AM. CCR2-mediated recruitment was not required for maintenance of AM numbers or for AM to develop the remodeled phenotype. IFN-γ signaling to AM drove very select aspects of the AM remodeled phenotype, only noted for MHC-II upregulation. The newly recruited and embryonic origin AM behaved similarly in regard to their surface marker remodeling after recovery from pneumococcal pneumonia. However, these subsets of AM differed in their phagocytic uptake of pneumococcus, and that difference was influenced by prior infection history.

Our study highlights different dynamics of Siglec F and MHC-II kinetics. The changes in Siglec F expression occurred within 1 day, suggesting a role for innate immune signaling. MHC-II intensity underwent slower changes that were amplified by a secondary challenge, suggesting a possible role for adaptive immune signaling. However, depletion of CD4^+^ cells did not affect Siglec F or MHC-II on the AM in pneumococcus-experienced lungs. These results contrast with resolution of adenovirus infections, in which CD8^+^ T cells are essential to the increased MHC-II on AM, specifically as sources of IFN-γ (22). CD4^+^ T cells were only one of many IFN-γ sources after pneumococcal infection. In addition to confirming neutrophils as providing IFN-γ during pneumococcal pneumonia (27, 28), our study revealed multiple additional cells as sources. Many cellular sources of IFN-γ may decrease the reliance on CD4^+^ cells for AM remodeling after pneumococcal infection.

Our results define a role of IFN-γ signaling in changing MHC-II intensity on AM in lungs with pneumococcal experience, and this increased MHC-II phenotype is directly dependent on IFN-γ ligand binding to its receptor on AM. These results support previous data establishing a role of IFN-γ in the remodeled AM phenotype that includes an increase in MHC-II (22). However, our data also demonstrate that most aspects of AM remodeling were independent of IFN-γ. This includes the decreased Siglec F surface levels on AM, as well as increased immunity in experienced lungs such as CD4^+^ T_RM_ cells, neutrophil recruitment, and bacterial elimination. We also found that phagocytosis, levels of numerous AM phagocytic and pattern-recognition receptors, and multiple lung cytokines were unchanged due to interruption of IFN-γ signaling. The increased MHC-II on AM surfaces stands out as a consistent and significant effect of interrupted IFN-γ signaling on AM remodeling. Mechanisms responsible for the decreased Siglec F levels on AM are yet to be determined, after recovery from pneumococcus or from any other pulmonary challenges.

The kinetics of AM loss and recovery after pneumococcal infection, combined with the surface marker changes over the time course, suggested a degree of cell turnover and replacement by AM of adult monocyte origin. The lineage-tracing mouse model confirmed this inference, shifting the pool of AM from ~15% adult HSC origin to ~80% adult HSC origin. These results support previous data highlighting the contributions of recruited monocytes to recovering AM numbers after pneumococcal pneumonia (13). Recruited monocytes contribute to the remodeled AM phenotypes defined by MHC-II and Siglec F after recovery from influenza infections (14) — but not after adenovirus infection (22) or after pneumococcal infections. With pneumococcal infection, CCR2 mediates the initial recruitment of Ly6C^+^ monocytes, but neither AM numbers nor phenotypes in the recovered lung depended upon this chemokine receptor. The numbers of AM in the recovered lungs of CCR2-deficient mice may result from monocytes recruited via CCR2-independent chemoattractants and/or local proliferation of AM. Influenza, adenovirus, and pneumococcus cause infections that result in varied patterns of lung recovery in mice, with different types of AM remodeling as discussed above in addition to differing degrees of epithelial abnormalities and of bronchus-associated tissue formation. The role of CCR2 in AM remodeling seems so far to be specific to recovery from influenza infections.

Beyond CCR2 independence, our data demonstrate that both the initial embryonic origin AM and the more newly recruited monocyte-derived AM subsets undergo similar remodeling after recovery from pneumococcal pneumonia. This substantiates the paradigm that local tissue microenvironment is the key determinant of macrophage phenotype (39–42). Prior studies comparing AM of adult HSC origin versus embryonic origin suggested they were largely similar (18, 19). Consistent with this, we found that MHC-II, Siglec F, and cytokine induction all similarly change due to prior history of pneumococcal infections in AM from either origin — embryonic or adult HSC. However, embryonically derived AM exhibit differing degrees of phagocytosis of pneumococcus compared with their recruited counterparts. Related to this, the AM of disparate origins display different surface levels of multiple receptors involved in recognizing and ingesting targets, including increases in CD93, TLR4, TLR2, and mannose receptor in the more recently recruited AM along with increased Dectin-1 in the embryonic origin AM. Experience changed the relative differences in pneumococcus phagocytosis by these AM subsets, without changing their relative levels of the receptors measured. Altogether, these data highlight the need to better understand how AM origin influences AM function, and when and how the influences of ontogeny on behavior are modified by prior infectious history.

A limitation of the present studies is that they were performed in mice. Myeloid cells including AM from lungs of humans and mice are similar but not identical when characterized at genome-wide transcriptome levels (43). Human AM and mouse AM respond similarly when their pneumococcus-induced transcriptomes are compared, and mouse AM responses to pneumococcus are even more human-like if the mice had previously experienced pneumococcal infections (21). Because mice that have recovered from pneumococcal pneumonia have multiple components of lung immunity that better resemble the human system (21, 23–26), there is ample support for the concept that prior experiences with respiratory infections are a driving feature of human lung immunology (6). However, the degree to which the biology detailed here applies to AM of other species, including humans, is unknown. Whether and how human AM phenotypes depend upon prior infection histories, and differences between AM of embryonic origin versus more recent recruits within the same human lung, remain important questions for further study.

Alveolar macrophages are different after recovery from pneumococcal pneumonia. These changes involve an element of cell replacement, with training that occurs in both the initial embryonic-origin AM, as well as the more newly recruited AM. How AM are remodeled after recovery from challenges is only beginning to be appreciated and needs to be defined and differentiated across infection settings. AM changes likely contribute to the improved defense against respiratory infection developing in childhood and preventing pneumonia in young adults; further studies should determine how aging influences remodeled AM phenotypes and may contribute to pneumonia in older subjects. Elucidating mechanisms underlying AM phenotypes will empower measuring and manipulating AM biology and development of future precision medicine approaches for countering pneumonia susceptibility.

## Methods

### Mice.

C57BL/6J, CCR2^–/–^ (44), and IFN-γ^–/–^ (45) mice were purchased from The Jackson Laboratory. Mice with a conditional mutation of IFNGR1 in CD11c cells were created at our mouse facilities by crossing CD11c-Cre mice (30) with IFNGR1-floxed mice (29) from The Jackson Laboratory. Flk-switch mice were initially generated by Thomas Boehm (Max-Planck Institute of Immunobiology and Epigenetics, Freiburg, Germany) (36–38). Age- and sex-matched littermate control mice were used for genetically engineered mice maintained in house; no effects of sex were noted for any of the remodeling outcomes measured. Both sexes were included in experiments, with the exception of those involving Flk-switch mice, in which males were studied to allow sex-matching since only males express the Cre transgene. All experiments were carried out on mice between 6 and 12 weeks of age. Mice were maintained in pathogen-free facilities of Boston University.

### Bacterial strains.

Sp19F (EF3030) was provided by Marc Lipsitch (Harvard Chan School of Public Health, Boston, Massachusetts, USA). *S*. *pneumoniae* serotype 3 (Sp3) was purchased from ATCC (catalog 6303). Bacteria were grown for 13–14 hours at 37°C in a humidified 5% CO_2_ environment on inverted tryptic soy agar plates containing 5% sheep blood before being suspended in antibiotic-free media for cell culture experiments or sterile saline for in vivo experiments. Bacterial inputs were confirmed using serial dilution CFU assays.

### Pneumococcal experience model.

Experienced mice and saline controls were generated by i.t. or i.n. infecting mice with 2 doses of Sp19F (strain EF3030) or saline, respectively, at a 1-week interval in order to generate effective heterotypic immunity (21, 23–26). All mice were allowed 4 weeks of rest to allow resolution of previous pneumonia and to restore homeostasis. Mice ushered through experience were either phenotyped at baseline or challenged with the more virulent pneumococcal strain Sp3 as described in figure legends.

### I.t. instillation model.

Mice were anesthetized by i.p. injection of ketamine (50 mg/kg) and xylazine (5 mg/kg). Mouse tracheas were surgically exposed and cannulated with an angiocatheter directed to the left bronchus. Mice were challenged with either *S*. *pneumoniae* EF3030 (Sp19F) or serotype 6303 (Sp3). Naive control mice received equal volumes of sterile saline. Serial instillations used the same procedures and sites for each instillation.

### Lung lobe homogenates.

Mice were euthanized at designated time points, and left lung lobes were harvested into 5 mL tubes and mechanically digested in sterile water containing 1× protease inhibitor (Roche, 11849300). Homogenates were either serially diluted in sterile saline to be plated on 5% sheep blood agar plates and incubated overnight at 37°C for measurement of bacterial burden, or they were mixed with cytokine lysis buffer and centrifuged at 15,000*g* for 20 minutes at 4°C for cytokine measurements.

### Broncho-alveolar lavage (BAL).

At indicated time points, mice were euthanized and exsanguinated, after which the right bronchus was closed by ligature. A catheter was inserted via the trachea, through which 5 boluses of 400 μL PBS were instilled and retrieved. BAL fluids were centrifuged at 300*g* for 5 minutes at 4°C, and supernatants were stored at –80°C. Pelleted cells were enumerated using LUNA-FL Dual Fluorescence Cell Counter (Logos Biosystems) and differentiated using cyto-centrifuge preparations stained with Diff-Quick (VWR).

### IFN-γ protein measurement.

IFN-γ concentrations were measured in BAL fluids using mouse IFN-γ ELISA duo sets (R&D Systems, DY485).

### SCS.

The lungs of euthanized mice were perfused, and left lung lobes were excised and collected into 3 mL RPMI-1640 containing 10% FBS. The lung lobes were minced with a razor blade while in a 1 mL digestion solution containing 1 mg/mL type II collagenase (Worthington Biochemicals), 150 μg/mL DNase, and 2.5 mM CaCl_2_. Volume was brought up to 5 mL of digestion solution and transferred into 50 mL conical tubes. Digested lungs were shaken at 37°C for an hour, after which the digestion solution was pushed through a 70 μm cell strainer (Thermo Fisher Scientific) to create SCS. Cells were pelleted via centrifugation (300*g* for 5 minutes at 4°C) and resuspended in 1 mL RBC lysis buffer (MilliporeSigma) for 2 minutes at room temperature. A volume of 10 mL PBS was added to each sample to stop the lysis, and cells were pelleted again by centrifugation (300*g* for 5 minutes at 4°C). SCS were counted using a Luna cell counter (Logo Biosystems). For experiments differentiating intra- and extravascular CD45 cells, an intravascular CD45.2-BUV737 or -FITC antibody (clone 104 BD Biosciences) was injected 3 minutes prior to euthanasia.

### Flow cytometry.

SCS were resuspended in media containing FACS solution (PBS, 2 mM Ethylenediaminetetraacetic acid and 0.5% FBS), Fc block (αCD16/CD32, Thermo Fisher Scientific), and Brilliant Stain buffer (BD Biosciences). The following fluorochrome-conjugated monoclonal antibodies were used to stain SCS: CD45-BV510, -FITC, or -PerCP-Cy5.5 (clone 30-F11, Thermo Fisher Scientific); CD64-PE or -APC (clone X54-5/7.1, BioLegend); Ly6C-eFluor 450 (clone HK1.4, Thermo Fisher); Ly6G-FITC (clone 1A8, BD Biosciences); MHC-II–PerCP–Cy5.5 or –BV421 (clone M5/114.15.2, BD Biosciences); Siglec F–APC/Cy7 (clone E50-2440, BD Biosciences); CD11b-BUV395 (clone M1/70, BD Biosciences); CD11c-PE/Cy7 or -BV421 (clone HL3, BD Biosciences); CD119-Biotin (clone GR20, BD Biosciences); IFN-γ–PE (clone XMG1.2, BioLegend); CD103 (clone M290, BD Biosciences); CD4-BV510 (clone GK1.5, BioLegend); CD8-APC/Cy7 (clone 53-6.7, BioLegend); CD3-FITC (clone 17A2, Thermo Fisher Scientific); CD19-BUV395 or -FITC (6D5, BioLegend); CD44-BV421(clone IM7, BD Biosciences); CD69-PE (clone H1.2F3, BioLegend); CD11a-APC (clone M17/4, Thermo Fisher Scientific); CD62L-PE/Cy7 (clone MEL-14, BioLegend); CD93-BV510 (clone 493, BD Biosciences); TLR2-APC (clone QA16A01, BioLegend); TLR4-PE/CY7(clone SA15-21, BioLegend); MMR-PerCP/Cy5.5(clone CO68C2, BioLegend); MSR-A-BUV395 (clone 268318, BD Biosciences); CLECL7a-BUV737 (clone 218820, BD Biosciences); CD45.2-BUV737 or -FITC (clone 104, BD Biosciences); and Streptavidin-PE (catalog 554061, BD Biosciences). Cells were stained with 7-AAD Viability Staining Solution (BioLegend). Unstained, single-stained and fluorescence-minus-one (FMO) controls were used for each analysis. All staining was performed on ice in the dark for 30 minutes. Stained cells were analyzed using a LSR-II (BD Biosciences) or Cytek Aurora and sorted using BD FACS Aria. Data were analyzed using FlowJo V10 software (BD Biosciences).

### Intracellular flow cytometry staining.

To collect cells containing synthesized proteins including those destined for secretion, SCS were plated in RPMI media containing Brefeldin A (BioLegend) and Monensin (BioLegend) solutions for 4 hours. These cells were collected and stained with a fixable viability dye, Fc blocked, and stained for extracellular receptors for 30 minutes. Cells were washed twice with FACS buffer and fixed (Intracellular Fixation & Permeabilization Buffer Set, eBioscience) for 20 minutes; they were then washed and resuspended overnight in FACS buffer. The following day, SCS were permeabilzed (Intracellular Fixation & Permeabilization Buffer Set, eBioscience), stained with IFN-γ–PE (clone XMG1.2 BioLegend) for 30 minutes, washed, and resuspended in FACS buffer. Unstained, single-stained, and FMO controls were used for each analysis. All staining was performed on ice in the dark for 30 minutes. Stained cells were analyzed using a LSR-II (BD Biosciences). Data were analyzed using FlowJo V10 software (BD Biosciences).

### Phagocytosis assays.

Sp3 were labeled with CellVue Claret dye or PKH67 for 3 minutes and washed thoroughly. Mice were challenged i.n. with fluorescently labeled Sp3. After 40 minutes, mice were euthanized and bronchoalveolar lavages were collected; cells were pelleted via centrifugation (300*g* for 5 minutes at 4°C), washed, and counted using a Luna cell counter (Logo biosystems). After blocking and labeling as described previously, cells were examined using the Cytek Aurora, and data were analyzed using FlowJo V10 software (BD Biosciences).

### CD4^+^ cell depletion studies.

Naive mice being ushered through pneumococcal experience were administered anti-CD4 monoclonal antibody (clone GK1.5) or IgG2b isotype control (BioXcell) via i.p. instillations (500 μg/100 μL) and i.n. instillations (100 μg/100 μL). The antibodies were delivered 1 day before and then both 1 day and 3 days after each of the 2 infections with Sp19F.

### Cell culture.

RAW264.7 cells were purchased from American Type Culture Collection (ATCC) and maintained in DMEM supplemented with 10% FBS and 1% penicillin/streptomycin (Thermo Fisher Scientific).

### Confocal imaging.

RAW 264.7 cells were seeded at 5 × 10^5^ cells/well in 35 mm glass-bottom culture dishes (Mat Tek); 2 hours later, DMEM was replaced with DMEM containing recombinant mouse IFN-γ (R&D systems) or vehicle overnight. The following day, cells were incubated with Sp3 in antibiotic-free DMEM media for 2 hours. Cells were washed with PBS and replaced with phenol red free DMEM (supplemented with 10% FBS + L-glutamine and sodium pyruvate) containing Hoechst (Invitrogen, 33342) and Cell Mask Green plasma membrane stain (Invitrogen, C37608) according to manufacturer recommendations and kept for 30 minutes at 37°C in a humidified atmosphere containing 5% CO_2_. Following that, cells were washed once with PBS and placed in phenol red free DMEM (supplemented with 10% FBS + L-glutamine and sodium pyruvate), and 1 drop/mL of NucRed Dead 647 Ready Probes Reagent (Molecular Probes, R37113) was added to measure cell membrane permeability. Confocal images were captured with a Zeiss LSM 710-Live Duo scan microscope with 20× objective. Images were randomly coded so that the investigator could score them while blinded to study group and processed using ImageJ software (NIH) and Zen imaging software.

### Lactate dehydrogenase assay.

RAW 264.7 cells were seeded at 5 × 10^5^ cells/well in 24-well plate. After 2 hours, DMEM was replaced with DMEM containing recombinant IFN-γ (R&D systems) or vehicle. The following day, cells were incubated with Sp3 in antibiotic-free DMEM for 2 hours, before media was replaced with complete DMEM containing antibiotics for an additional 4 hours of culture. Supernatants were transferred into 96-well plates, and LDH assays (Promega) were performed as described by the manufacturer.

### Measurement of mRNA.

Sorted alveolar macrophages were centrifuged at 300*g* for 5 minutes at 4°C and resuspended in RLT lysis buffer. RNA was processed using Qiagen micro-kits (Qiagen RNeasy Micro kit), as described by the manufacturer.

### Statistics.

Statistical analyses were performed using GraphPad Prism software (Version 8.4.3). Specific statistical analysis used for each dataset are described in figure legends. Unpaired data were analyzed using parametric tests, with 2 groups compared using the 2-tailed Student’s *t* test, and more than 2 groups were compared using a 1-way or 2-way ANOVA followed by Tukey’s or Dunnett’s for multiple comparisons. For data that failed normality tests, Mann-Whitney *U* tests or Kruskal-Wallis followed by Dunn’s multiple-comparison tests were used to compare groups. Paired data were analyzed using the paired *t* test analysis. Differences were considered significant if *P* < 0.05.

### Study approval.

All animal protocols were approved by the Boston University IACUC.

## Author contributions

EIA and JPM conceived of and designed the experiments. ACB, AG, ECF, MRJ, LJQ, and JPM contributed to experimental design, interpretation of results, and mentorship of those performing experiments. EIA, ATS, KAB, NSE, IMCM, CLDA, EN, CVO, WNG, FTK, AKW, and ACB performed experiments. All authors discussed data and interpretations. EIA and JPM constructed the manuscript and wrote initial drafts. JPM supervised the project.

## Version 1 02/08/2022

In-Press Preview

## Version 2 03/08/2022

Electronic publication
